# Glucocorticoids as adjunctive therapy for severe myelosuppression induced by combined immune checkpoint inhibitor and chemotherapy: a case report and literature review

**DOI:** 10.3389/fonc.2026.1801698

**Published:** 2026-05-01

**Authors:** Shimin Tang, Na Li, Ji Ma

**Affiliations:** Department: Second Ward, Oncology Center, Suining Central Hospital, Suining, Sichuan, China

**Keywords:** chemotherapy, glucocorticoids, immune checkpoint inhibitor (ICI), lung neoplasms, myelosuppression

## Abstract

**Objective:**

We conducted a retrospective case series of two lung cancer patients treated at Suining Central Hospital (January–March 2025) who developed severe myelosuppression after immune checkpoint inhibitor (ICI) –chemotherapy combination therapy, to characterize its clinical characteristics and management.

**Methods:**

A retrospective analysis was conducted. Severe myelosuppression (grade IV, CTCAE 5.0) and pancytopenia (trilineage cytopenia) were clearly defined. Immune-related myelosuppression was diagnosed by exclusion of other etiologies. Clinical data and outcomes were analyzed, and a literature review was performed.

**Results:**

Both patients with severe myelosuppression after ICI–chemotherapy combination therapy achieved complete hematologic recovery with high-dose glucocorticoids plus standard supportive care.

**Conclusion:**

Although chemotherapy is the main cause of myelosuppression, severe myelosuppression following ICI–chemotherapy is multifactorial. Clinicians should perform prompt differential diagnosis and consider an immune-related mechanism. Early glucocorticoids are essential if ICI-related toxicity is suspected. As a small retrospective study, further large-scale trials are needed to validate optimal glucocorticoid dosing, duration, and individualized strategies.

## 1 Introduction

ICI alleviate immunosuppression, activate T lymphocytes, and restore immune surveillance by blocking inhibitory pathways such as cytotoxic T-lymphocyte-associated antigen 4 (CTLA-4) or programmed death-1 (PD-1), thereby serving as pivotal antitumor therapies (1). With the widespread clinical application of ICI, immune-related adverse events (irAEs) have become increasingly prominent. However, ICI-related myelosuppression remains rarely reported. Data from the RATIONALE-305 study showed that, compared with chemotherapy alone, the tislelizumab plus chemotherapy group did not exhibit a significant increase in myelosuppression-related adverse events (2). Liu DY et al. (3) demonstrated by multivariate analysis that leukopenia after chemotherapy was significantly associated with concurrent ICI treatment (P = 0.007). Although ICI-induced myelosuppression is mostly mild, evidence remains insufficient regarding whether combination with chemotherapy may exacerbate myelosuppression.

Myelosuppression is a common chemotherapy-related adverse event. Severe myelosuppression can cause chemotherapy dose reductions or delays, induce life-threatening complications (e.g., infection, bleeding), impair treatment efficacy and patient quality of life, and increase economic burden, all of which adversely affect prognosis. However, severe myelosuppression complicated by pancytopenia following ICI–chemotherapy combination therapy is relatively rare. By analyzing two such cases, this study aims to enhance clinicians’ awareness of this severe adverse event and provide diagnostic and therapeutic references, which is reported herein.

## 2 Case presentation

### 2.1 Case 1

#### 2.1.1 Baseline data

A 75-year-old female with Eastern Cooperative Oncology Group (ECOG) performance status 1. Height: 143 cm, weight: 45 kg, body surface area (BSA): 1.30 m². She was admitted on January 21, 2025, due to diarrhea and anorexia for 2 days, 6 months after diagnosis of small cell lung cancer (SCLC) of the right lung. In July 2024, a right upper lobe and hilar mass was detected on routine examination. Bronchoscopy with biopsy on July 4, 2024, confirmed small cell carcinoma (right main bronchus). Imaging staged the disease as limited-stage cT4N2M0 (IIIB). She received concurrent chemoradiotherapy with etoposide plus carboplatin (EC). Radiotherapy: 95% PTV, total 45 Gy in 30 fractions (1.5 Gy twice daily).Chest and whole-abdominal contrast-enhanced CT on January 7, 2025, showed disease progression. On January 8, 2025, she received one cycle of tislelizumab 200 mg day 1 plus irinotecan 130 mg days 1 and 8 (q3w).On January 19, 2025, watery diarrhea (3–5 times/day) developed, partially relieved by montmorillonite and loperamide (2–3 times/day), accompanied by anorexia and fatigue, without fever, chills, cough, or expectoration.

Physical examination showed diminished breath sounds over the right lung, with no rales bilaterally. The abdomen was mildly tender without rebound or guarding. Bowel sounds were present at 8 beats/min. No shifting dullness or lower extremity edema was noted.

Laboratory Examinations: Complete blood count (CBC) on January 21, 2025, revealed: white blood cell (WBC) 0.3×10^9/L, neutrophil count (NEUT #) 0.02×10^9/L, hemoglobin (HGB) 92g/L, and platelet (PLT) 46×10^9/L.

Diagnoses: (1). Myelosuppression (IV°), (2). Leukopenia (IV°), (3). Neutropenia (IV°), (4). Thrombocytopenia (III°), (5). Moderate anemia, (6). Small cell carcinoma of the upper lobe hilum of the right lung (limited stage, cT4N2M0, Stage IIIB), (7). Functional diarrhea.

#### 2.1.2 Treatment course

**Figure 1:**
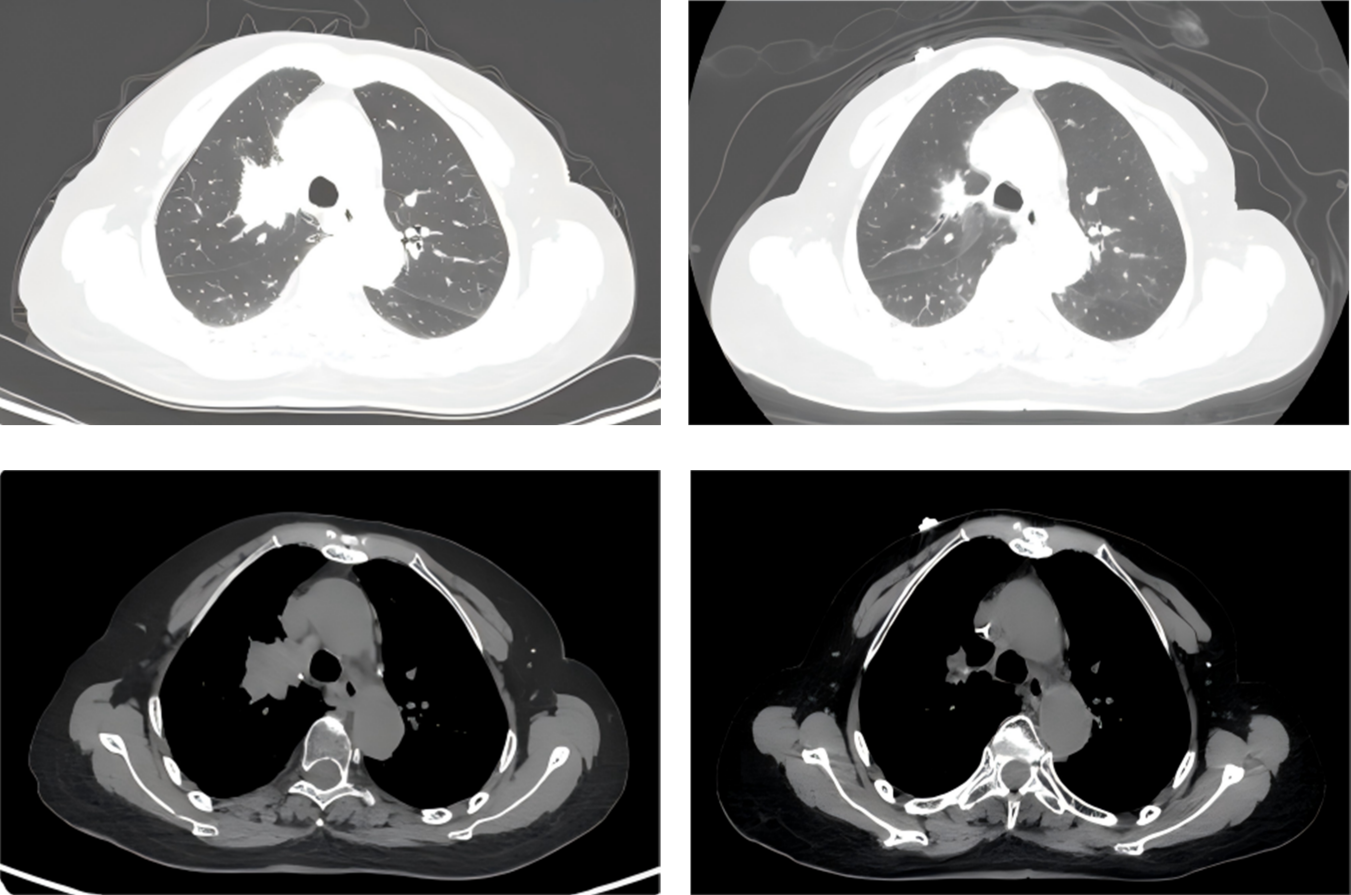
Eight days after ICI–chemotherapy, the right lung lesion showed marked reduction, achieving PR.

**Figure 2:**
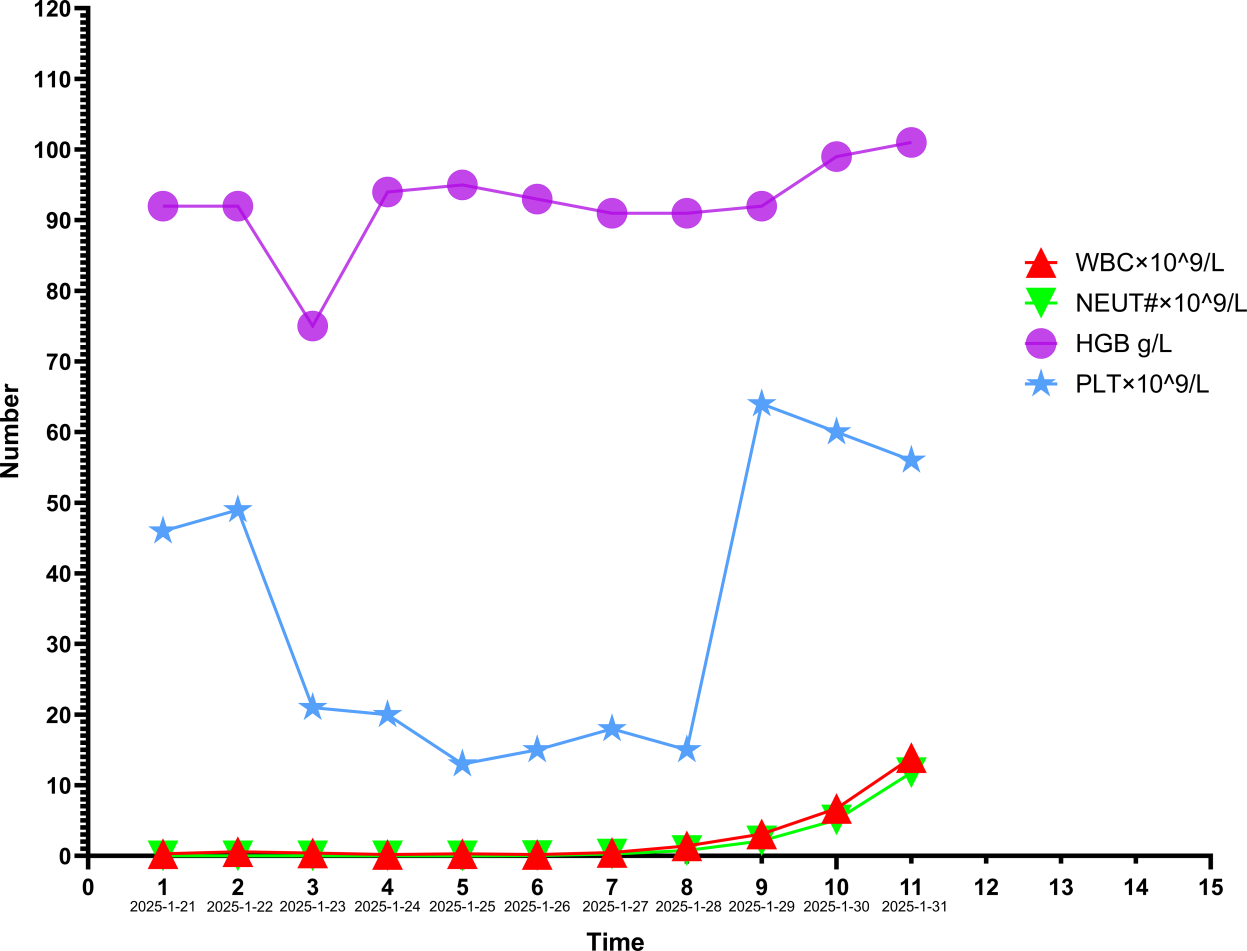
CBC changes and interventions in patient 1.From January 21, 2025 to January 25, 2025: Treatment regimen: rhG-CSF, TPO, EPO, imipenem/cilastatin, antidiarrheals, and intravenous nutritional support;From January 26, 2025 to January 30, 2025: Treatment regimen: methylprednisolone, rhG-CSF, TPO, EPO, vancomycin, imipenem/cilastatin, and intravenous nutrition. Platelet transfusion was additionally administered on January 28, 2025;On January 31, 2025: Treatment regimen: voriconazole, TPO, tapered methylprednisolone, piperacillin-tazobactam, and vancomycin.

1. January 21–23, 2025: The patient presented with abdominal pain, watery diarrhea (2–3 episodes/day), anorexia, and fatigue, without fever, chills, cough, or sputum. Treatments administered: ① recombinant human granulocyte colony-stimulating factor injection (rhG-CSF) 300 μg ih qd; ② Recombinant human thrombopoietin injection (TPO) 15 000 U ih qd; ③Erythropoietin (EPO) 3000 U ih qod; ④ Imipenem/cilastatin 1 g iv q8h(prophylactic broad-spectrum antibiotic use); ⑤ Loperamide and montmorillonite; ⑥ Intravenous nutritional support. January 24–26, 2025: The patient developed fever (peak 39.0 °C), mild cough with white mucoid sputum, in addition to persistent abdominal pain, watery diarrhea (1–2 episodes/day), anorexia, and fatigue. The above regimen was continued.
2. Follow-up CBC on January 26, 2025: WBC 0.2×10^9^/L, NEUT# 0.07×10^9^/L, HGB 93 g/L, PLT 15×10^9^/L. Liver and renal function were normal. Coagulation tests showed fibrinogen 3.71 g/L and serum ferritin 1490 μg/L. Chest and whole-abdomen non-contrast CT on the same day, compared with January 6, 2025, showed heterogeneous opacities in both lungs. The right upper lobe-hilar soft-tissue mass measured 2.0 cm × 1.8 cm (vs. 3.5 cm × 3.6 cm), indicating marked regression. Bilateral scattered fibrous foci and chronic inflammatory changes were decreased. Per RECIST 1.1, the response was graded as partial response (PR) (**Figure 1**). Given strong suspicion of immune-related myelosuppression, methylprednisolone 120 mg iv qd and vancomycin 0.5 g iv q8h were initiated. Antinuclear antibodies (ANA) and extractable nuclear antigens (ENA) were not tested due to critical illness and family refusal of autoantibody screening, bone marrow aspiration, and biopsy.
3. Follow-up CBC on January 28, 2025: WBC 1.4×10^9^/L, NEUT# 0.78×10^9^/L, HGB 91 g/L, PLT 15×10^9^/L. One therapeutic unit of platelets was transfused, and the remaining regimen was continued unchanged.
4. On January 31, 2025, the patient’s appetite improved, with fever and diarrhea resolved and fatigue alleviated. CBC: WBC 13.9×10^9^/L, NEUT# 11.8×10^9^/L, HGB 101 g/L, PLT 56×10^9^/L. Galactomannan (GM): 1.97 μg/L (positive, cutoff <0.25 μg/L); (1,3)-β-D-glucan: 195.43 pg/mL (positive, cutoff <60 pg/mL). Stool testing showed 4+ fungi. G-CSF and EPO were discontinued; TPO was continued. Methylprednisolone was tapered gradually. Antibiotics were de-escalated to piperacillin-tazobactam 4.5 g iv q12h plus vancomycin 0.5 g iv q8h. Oral voriconazole 100 mg q12h was added for antifungal coverage. The patient improved and was discharged. Hematologic trends and interventions are shown in **Figure 2**.

### 2.2 Case 2

#### 2.2.1 Baseline data

A 59-year-old female, ECOG PS 1, height 155 cm, weight 45 kg, BSA 1.37 m². She was admitted on March 9, 2025, with newly detected myelosuppression (1 day) and a 2-year history of lung cancer. A right upper lobe mass was found incidentally in March 2022; percutaneous lung biopsy on March 29, 2022, confirmed adenocarcinoma. Whole-body bone scan on March 30, 2022, showed L3 mixed osteolytic/osteoblastic destruction with pathological fracture, consistent with bone metastasis. Radiotherapy (95% PTV, 30 Gy in 10 fractions of 3.0 Gy) was delivered on April 3, 2022. Genetic testing revealed EGFR exon 21 L858R mutation. Disease progressed despite third-generation EGFR-TKI. On March 1, 2025, she received one cycle of ivonescimab 900 mg + 15% dose-reduced pemetrexed 580 mg + carboplatin 300 mg(AC chemotherapy) (d1, q3w). Pegfilgrastim was given within 24 hours for primary prophylaxis.

CBC on March 9, 2025: WBC 0.94×10^9^/L, NEUT# 0.57×10^9^/L, HGB 92 g/L, PLT 25×10^9^/L. No fever, chills, cough, or sputum was reported.

Physical examination: unremarkable.

Diagnoses: (1) Myelosuppression (IV°), (2) Leukopenia (IV°), (3) Neutropenia (III°), (4) Thrombocytopenia (III°), (5) Mild anemia, (6) Right lung adenocarcinoma with bone metastasis (cT3N0M1, Stage IV, EGFR exon 21 L858R).

**Figure 3:**
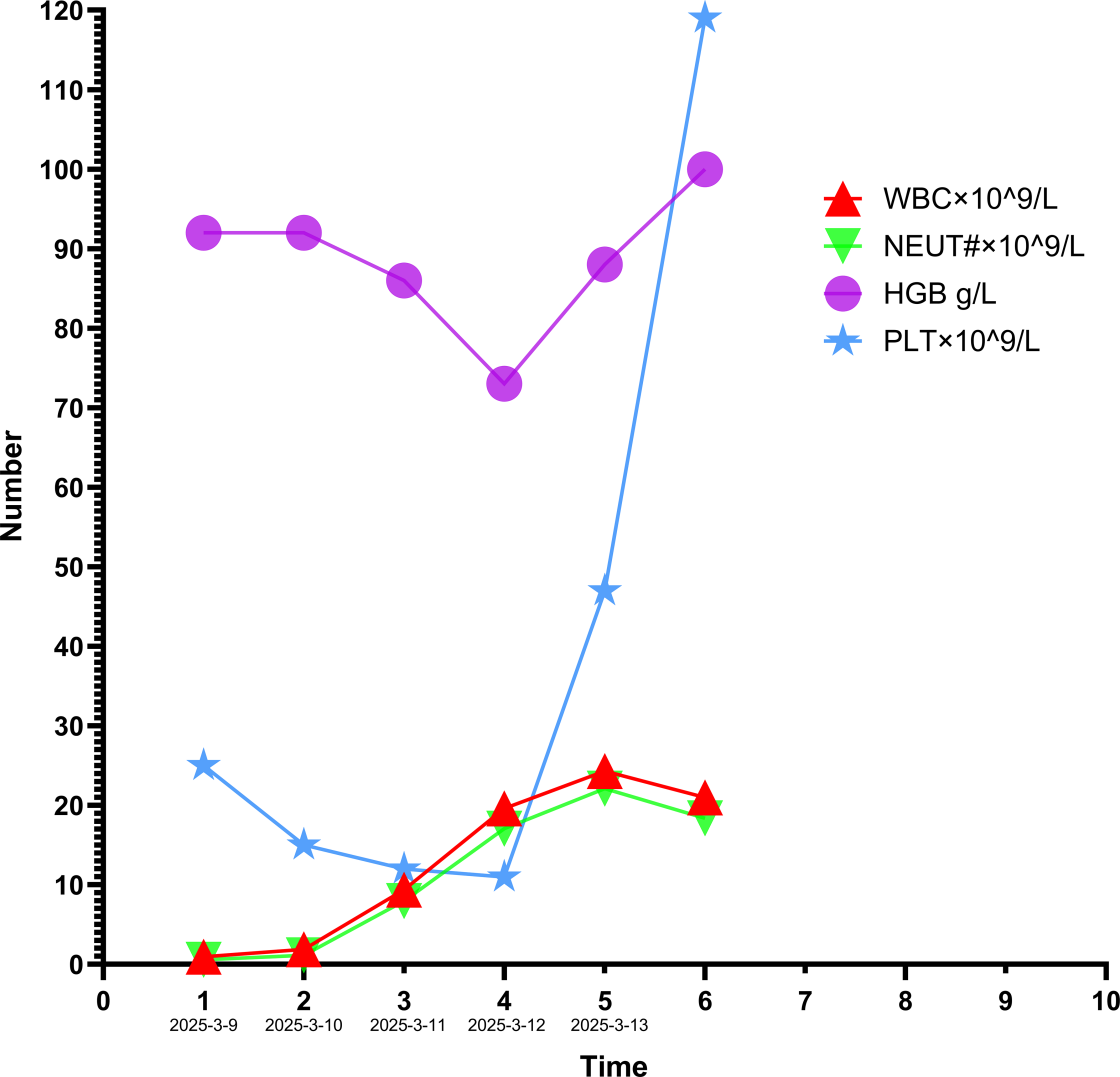
CBC changes and interventions in patient 2.From March 9, 2025 to March 12, 2025: Treatment regimen: rhG-CSF, TPO, EPO, antibiotics, and methylprednisolone;On March 13, 2025: Treatment regimen: TPO, EPO, and tapered methylprednisolone.

1.2.2 Treatment Course:

1. On March 9, 2025, the following treatments were initiated:① rhG-CSF 300 μg ih qd;② TPO 15 000 U ih qd;③ EPO 3000 U ih qod;④ Methylprednisolone 100 mg iv qd;⑤ Piperacillin-tazobactam 4.5 g iv q12h(prophylactic broad-spectrum antibiotic use).
2. On March 13, 2025, follow-up CBC: WBC 24.30×10^9^/L, NEUT# 22.11×10^9^/L, PLT 47×10^9^/L.TPO and EPO were continued; methylprednisolone was tapered gradually. The patient improved and was discharged. Hematologic trends and interventions are shown in **Figure 3**. Clinical characteristics and treatment courses of both patients are summarized in **Table 1**.

**Table 1:** Summary of therapeutic data for the two patients.

Patient number	Age(years)	Gender	Medication	Medication cycle	Myelosuppression treatment regimen	Prognosis	Evaluation of association with ICI
1	75	Female	Tislelizumab + Irinotecan	1	Methylprednisolone + Conventional Treatment	Complete Recovery	Highly Probable
2	59	Female	Ivosidenib + Pemetrexed + Carboplatin	1	Methylprednisolone + Conventional Treatment	Complete Recovery	Highly Probable

## 3 Discussion

Bone marrow comprises hematopoietic cells and their microenvironment, which act synergistically under genetic regulation to maintain hematopoiesis (4). Chemotherapy damages hematopoietic stem cells (HSCs), reducing self-renewal and inducing senescence, thereby impairing long-term hematopoiesis. It also disrupts mitochondrial function and oxidative balance, promoting HSC damage and apoptosis (5). Elevated TNF-α, TGF-β, and IFN-γ further suppress hematopoiesis (6). The pathogenesis of ICI-related myelosuppression is multifactorial and incompletely defined. ICI enhance T and NK cell cytotoxicity, leading to immune attack on marrow precursors and trilineage failure, which may be persistent or life-threatening (7). Myelosuppression from ICI–chemotherapy is more complex than with either agent alone and cannot be fully attributed to ICI. Chemotherapy remains the primary cause, while ICI likely exacerbate toxicity via immune-mediated damage to the hematopoietic microenvironment and cells, with synergistic effects. This hypothesis requires further validation.

Wang Y et al. (8) reported 6.1% (5/82) of irAE-related deaths were due to hematologic toxicities (fourth leading cause, after respiratory failure, cardiovascular events, and infection) in 20,128 patients. Kramer R et al. (9) identified hematologic irAEs in 0.6% (50/7,626) of ICI-treated patients: thrombocytopenia and leukopenia each 34% (17/50), anemia 28% (14/50); 1 patient died of agranulocytosis. Mao YY et al. (10) showed grade ≥3 myelosuppression was 4.88% with ICI monotherapy vs. 12.86% with ICI–chemotherapy in elderly advanced NSCLC. Kanbour A et al. (11) reported pancytopenia following ICI in a patient with MSI-H endometrial cancer.

In this report, Case 1 received tislelizumab plus irinotecan. On post-therapy day 6, pancytopenia developed (grade 4 leukopenia/neutropenia, grade 3 thrombocytopenia). Standard severe chemotherapy-induced myelosuppression management was initiated promptly; however, after 6 days, leukopenia/neutropenia persisted at grade 4 and thrombocytopenia worsened to grade 4.Although delayed effects of rhG-CSF cannot be excluded, high-dose methylprednisolone led to rapid, supra-physiologic increases in WBC and neutrophil counts—exceeding the typical efficacy and onset of rhG-CSF alone—suggesting a core role of glucocorticoids in immune-related myelosuppression. Concurrently, follow-up CT showed rapid tumor regression (PR). A multidisciplinary team (MDT) considered differential diagnosis of hemophagocytic lymphohistiocytosis (HLH), a rare hyperinflammatory syndrome from excessive cytokine release. A retrospective study of 61 malignancy-associated HLH (MA-HLH) cases found only 9.8% arose from solid tumors (12). MA-HLH was not supported due to critical illness and family refusal of further workup (NK cell activity, bone marrow aspiration/biopsy, plasma sCD25). Combined with rapid tumor regression, the MDT concluded severe myelosuppression secondary to ICI–chemotherapy.

ICI-induced hematologic toxicities are uncommon, but higher rates have been reported with tislelizumab (13, 14). Wang Y et al. (15) retrospectively reviewed 79 patients treated with tislelizumab; hematologic adverse events were the most frequent (32.91%), mainly neutropenia, leukopenia, thrombocytopenia, and anemia, including 21 grade ≥3 events. Per the 2022 ESMO irAE guidelines, methylprednisolone is recommended for grade 3–4 myelosuppression (16). High-dose methylprednisolone resulted in rapid leukocyte and neutrophil recovery in our patient.

Patient 2 received ivonescimab plus AC chemotherapy. Per 2025 Chinese Society of Clinical Oncology (CSCO) guidelines, AC is low-risk for myelosuppression (17). A 15% dose reduction and pegfilgrastim prophylaxis were administered. On day 9 post-treatment, grade 4 leukopenia, grade 3 neutropenia, and thrombocytopenia developed, consistent with ICI-related myelosuppression. Ivonescimab is a PD-1/VEGF-A bispecific antibody. In the HARMONi-A study (18), grade ≥3 TRAEs occurred in 61.5% of patients receiving ivonescimab+AC, with neutropenia (31.1%), leukopenia (20.5%), thrombocytopenia (16.1%), and anemia (15.5%) being most common. High-dose methylprednisolone for 4 days, combined with standard supportive care, led to rapid trilineage recovery, confirming the efficacy of glucocorticoids in ICI-induced severe myelosuppression. In ICI–chemotherapy-related severe myelosuppression, other etiologies (hematologic disease, infection, chemotherapy alone) must first be excluded. Glucocorticoids are central to management, with potent anti-inflammatory and immunosuppressive effects. G-CSF, transfusion, and growth factors alone often yield suboptimal responses due to immune-mediated marrow microenvironment injury. Although glucocorticoids increase infection risk, they significantly improve outcomes when combined with standard measures. Patient 1 responded poorly to initial supportive care but recovered rapidly after methylprednisolone. Patient 2 received early glucocorticoids with prompt resolution. Thus, methylprednisolone plus G-CSF is recommended for grade 3–4 ICI-related neutropenia, with antimicrobial prophylaxis and nutritional support. The American Society of Clinica Oncology (ASCO) guidelines (19) recommend glucocorticoids for ICI-related anemia (Hb <100 g/L). ICI-related thrombocytopenia is managed like primary ITP, with glucocorticoids as first-line (20). Unlike lineage-specific growth factors, glucocorticoids simultaneously restore trilineage hematopoiesis in immune-mediated pancytopenia. Urgent diagnosis and early glucocorticoid initiation are critical for survival.

Limitations:① Small retrospective case series (n=2), limiting generalizability.② ICI and chemotherapy contributions to myelosuppression cannot be fully distinguished; synergistic mechanism remains unclear.③ Incomplete workup (ANA, ENA, bone marrow aspiration/biopsy) lacking direct evidence of immune-related myelosuppression.④ Optimal glucocorticoid dose, duration, and tapering schedule not defined.

## 4 Conclusion

In conclusion, severe myelosuppression following ICI–chemotherapy requires early assessment for glucocorticoid therapy alongside standard supportive care. This study is limited to two cases with low-level evidence; glucocorticoid use was empirical. Further large-scale trials are needed to define optimal glucocorticoid dosing, duration, and personalized regimens to improve safety and efficacy.

## Data Availability

The original contributions presented in the study are included in the article/supplementary material. Further inquiries can be directed to the corresponding author.
